# Extended homozygosity is not usually due to cytogenetic abnormality

**DOI:** 10.1186/1471-2156-8-67

**Published:** 2007-10-04

**Authors:** David Curtis

**Affiliations:** 1 Academic Centre for Psychiatry, St Bartholomew's and Royal London School of Medicine and Dentistry, Royal London Hospital, Whitechapel, London E1 1BB, UK

## Abstract

**Background:**

Previous studies have reported frequent stretches of homozygosity in human subjects but have failed to clarify whether these are due to cytogenetic abnormalities or to autozygosity.

**Methods:**

Trios which had been typed for closely spaced SNPs spanning the genome were studied. Stretches of extended homozygosity were identified in the child members, as were occasions on which the child had been genotyped as not inheriting one parental allele. The number of times such transmission errors occurred within regions of extended homozygosity was compared with the chance expectation.

**Results:**

Transmission errors occurred more rarely in regions of extended homozygosity than would be expected by chance.

**Discussion:**

Regions of extended homozygosity are not generally due to cytogenetic abnormalities such as uniparental isodisomy. They reflect the Mendelian inheritance of haplotypes from a common ancestor. This may have implications for mapping disease genes.

## Background

Two recent reports describe the extent to which regions of extended homozygosity can be found in human subjects genotyped with large numbers of closely spaced single nucleotide polymorphism (SNP) markers [1,2]. The frequency and length of these stretches may have been surprising to many readers. The first study reported 1393 tracts over 1 Mb in length among 209 subjects and the second found tracts extending over 5 Mb being present in 26 out of 272 subjects. Since the samples used were from apparently unrelated subjects in outbred populations one might not expect to find such regions by chance. The authors of the first study thought that the tracts probably represented ancestral haplotypes but could not rule out deletions or uniparental isodisomy as alternative explanations. Likewise, the authors of the second study proposed that the finding represented "chance meiotic events in consanguinous parents". Through resampling they were able to rule out the finding as being due to an artefact of the cell immortalisation process. Through examination of the hybridization intensity of each SNP they were able to estimate copy number and hence to identify a number of structural genomic variants, which meant they could exclude the observed homozygosity as being due to deletions. However this would still not exclude the possibility that uniparental isodisomy had occurred, this being the other form of cytogenetic abnormality which can be a cause of extended homozygosity. They did note that subjects with one region of extended homozygosity were more likely to have an additional region than would be expected by chance and argued that this supported the hypothesis that such regions were due to parental consanguinity. However an alternative hypothesis would be that some internal or external factor, for example a predisposition to mitotic errors or exposure to a mutagenic agent, could produce numerous regions of uniparental isodisomy in the same subject. Both studies were carried out on unrelated subjects and in such a sample it is hard to see how one could definitively rule this out such explanations.

An earlier study had used related subjects [3], consisting of 8 of the reference pedigrees from the Centre d'Etude du Polymorphisme Humain (CEPH) [4]. These were genotyped with 8000 short tandem repeat markers. The authors identified numerous long homozygous segments. These were compatible with Mendelian transmission, indicating that they were indeed a result of autozygosity, that is, the subject receiving a copy of the same ancestral haplotype from each parent. Thus these stretches of homozygosity did not appear to result from cytogenetic abnormalities. However a relatively small number of markers was used yielding an average inter-marker spacing of slightly less than 0.5 cM. With this marker density it would be difficult to distinguish whether some relatively short stretches of homozygosity might or might not be due to mechanisms such as uniparental isodisomy.

Here, I present the results of an investigation of regions of extended homozygosity detected by densely spaced SNPs genotyped in a sample of CEPH trios. If such regions were the result of cytogenetic abnormalities they should be detectable as departures from Mendelian transmission.

## Method

Samples genotyped using the Affymetrix 500 K chip set are available from the Affymetrix website [5]. Genotypes called using the DM algorithm are available for relatively large numbers of subjects. However preliminary studies revealed that this algorithm gave much higher rates of homozygosity, and longer and more frequent homozygous tracts, than the BRLMM algorithm [6]. Hence I decided to use only subjects for whom genotypes called using this latter algorithm were available. At the time the project was carried out these consisted of a small sample of ten subjects and their parents from ten different European (CEU) CEPH pedigrees whose genotypes could be downloaded from the website. (Subsequently, BRLMM genotypes have been made available for the whole HapMap sample.) The genotypes were used as provided from the site without any further quality control checks being applied.

In order to detect regions of homozygosity I looked for stretches in which there were at least ten contiguous markers which were homozygous. Interspersed markers with missing genotypes were ignored but the tract could not contain any markers called as heterozygous. The distance between the first and last marker had to reach 1 Mb or 5 Mb, according to the required tract length.

A number of different types of transmission error could be detected. For the purpose of the present analysis transmission errors were characterised on the premise that genotypes called assuming the presence of two alleles might actually reflect a different genotype if there were a cytogenetic abnormality, so that for example if a deletion had occurred then genotype A_ might be called as AA and if a trisomy were present genotype AAB might be called as AB. I identified "gained allele" errors when the child had an allele which neither parent possessed. However in order to look for transmission errors which might reflect the presence of either a deletion or uniparental isodisomy I counted as "lost allele" errors those in which one parent was AA, the other BB and the child either AA or BB.

I compared the observed number of "lost allele" errors occurring in regions of extended homozyogity to the number one would expect given the proportion of the markers falling within these regions in each subject. For simplicity, only autosomal chromosomes were studied. Markers on these chromosomes had an average spacing of 5.8 kb.

## Results

Among the ten subjects there were 263 regions in which ten or more contiguous markers were homozygous and which extended over 1 Mb or more. The fewest number of such regions found in one subject was 11 and the largest 39. The fraction of all autosomal markers falling within these regions in each subject ranged from 0.0017 to 0.0098, the average being 0.0060. Of these 263, there were 30 regions which were homozygous over 5 Mb or more. Only one subject had none while the number in the rest ranged from 1 to 6. Likewise the proportion of the autosomal markers involved ranged from 0.0 to 0.0011, averaging 0.00036.

With respect to transmissions to these subjects from their parents, it was possible to identify 4117 "lost allele" transmissions, representing a detectable error rate of 0.0084 out of a total of 4900320 child genotypes.

Table 1 shows the distribution of "lost allele" transmission errors within and outside regions of extended homozygosity of 1 Mb along with the numbers expected to be found given the proportions of markers which fell within such regions. In all cases the observed number of errors within a region of extended homozygosity is less than the expected number. Although only a small number of errors are expected to occur within such regions in each individual, if we total over all individuals then we obtain an observed count of 6 errors compared with an expected count of 20.2 and if we carry out a chi-squared test we find that there are significantly fewer "loss of allele" errors found with in regions of extended homozygosity than we would expect by chance, with p = 0.0015. No such transmission errors were found within regions of extended homozygosity of 5 Mb or more.

**Table 1:** Number of transmission errors occurring within and outside regions of extended homozygosity > 1 Mb compared with chance expectation

Subject	Observed number of "lost allele" errors related to regions of homozygosity of > 1 Mb		Proportion of markers falling within regions	Expected number of errors to be related to regions of homozygosity of > 1 Mb	
	Within regions	Outside regions		Within regions	Outside regions

1	0	239	0.0096	2.3	236.7
2	0	245	0.0094	2.3	242.7
3	3	274	0.0083	2.3	274.7
4	1	261	0.0092	2.4	259.6
5	1	244	0.0073	1.8	243.2
6	1	605	0.0068	4.1	601.9
7	0	597	0.0020	1.2	595.8
8	0	488	0.0025	1.2	486.8
9	0	602	0.0017	1.0	601.0
10	0	556	0.0029	1.6	554.4

Total*	6	4111	0.0049	20.2	4096.8

*Chi-squared = 10.0, 1 df, p = 0.0015.

## Discussion

Although based on a small sample, these results conclusively demonstrate that regions of extended homozygosity are not usually due to uniparental isodisomy. If they were, we would expect that within these regions on many occasions an allele which was homozygous in a parent would fail to appear in a child. In fact, although a small number of such transmission errors do occur, they are observed more frequently outside regions of extended homozygosity than within them. It is somewhat striking that no errors were observed within the 5 Mb regions, suggesting that even these long stretches of homozygosity reflect autozygosity rather than cytogenetic abnormality and implying that there are some very long haplotypes which are not uncommon in this outbred population.

A more sophisticated approach to the analysis might have included consideration of whether transmission errors tended to occur in consecutive markers. One would expect this to happen if a cytogenetic abnormality occurred. On the other hand, only a small proportion of errors are actually detectable because both parents must be homozygous for different alleles so that what one might expect to detect would be loose groupings of errors rather than strings of errors in consecutive markers. In any event, the results obtained from the crude method applied here seem to be conclusive in terms of the general finding although they do not exclude the possibility that a small proportion of the homozygous regions might be due to cytogenetic abnormalities.

The findings seem unlikely to be due to some artefact around the genotype calling process or related error-checking because there would be no differential effect within regions of extended homozygosity. The reason for the relative paucity of transmission errors appearing within these regions is a matter for speculation. It may be that markers within these regions are more likely to have low minor allele frequencies meaning that it would be relatively unlikely for a child and parent to be called as having different homozygous genotypes.

## Conclusion

These results demonstrate that while it is fairly common for regions of homozygosity to extend over 1 Mb this is usually due to segments of such length being inherited from a common ancestor rather than being due to deletion or uniparental isodisomy. As pointed out previously [1,2], this will have implications for the ability to map diseases using association studies.

## Competing interests

The author(s) declares that there are no competing interests.
